# Dysbiosis of saliva microbiome in patients with oral lichen planus

**DOI:** 10.1186/s12866-020-01733-7

**Published:** 2020-04-03

**Authors:** Fei Yan Yu, Qian Qian Wang, Miao Li, Ya-Hsin Cheng, Yi-Shing Lisa Cheng, Yu Zhou, Xi Yang, Fang Zhang, Xuejun Ge, Bin Zhao, Xiu Yun Ren

**Affiliations:** 1 Department of Oral Medicine, Shanxi Medical University School and Hospital of Stomatology, Taiyuan, 030001 Shanxi China; 2 Department of Periodontology, Shanxi Medical University School and Hospital of Stomatology, No. 63, New South Road, Yingze District, Taiyuan, Shanxi 030001 People’s Republic of China; 3 Department of Physiology, School of Medicine, China Medical University, Taichung, Taiwan; 4 Department of Diagnostic Sciences, Texas A & M University College of Dentistry, Dallas, TX USA; 5 Shanxi Medical University School and Hospital of Stomatology, Taiyuan, 030001 Shanxi China

**Keywords:** Oral lichen planus, Salivary microbiome, 16S rDNA, High-throughput sequencing

## Abstract

**Background:**

Oral microbiota is not only important for maintaining oral health but also plays a role in various oral diseases. However, studies regarding microbiome changes in oral lichen planus (OLP) are very limited. To the best of our knowledge, there has been only two studies investigating salivary microbiome changes in OLP. Therefore, the purpose of this study was to identify the characteristic microbial profile in the saliva of OLP patients, with or without erosive lesions, and compare that with recurrent aphthous ulcer (RAU), a common oral immunological disorder that also shows multiple erosive/ulcerative lesions. Whole saliva samples were collected from 20 patients with OLP (erosive E, n = 10 and non-erosive NE, n = 10), 10 patients with RAU (U) and 10 healthy controls (C). DNA was extracted from the saliva samples, and the 16S rDNA gene V4 hypervariable region was analyzed using Illumina sequencing.

**Results:**

We obtained 4949 operational taxonomic units (OTUs) from the V4 region in all saliva samples. Community composition analysis showed a clear decreased relative abundance of genera *Streptococcus* and *Sphingomonas* in saliva from RAU patients when compared to the other three groups. Relative abundance of *Lautropia* and *Gemella* were higher in E group, whereas relative abundance of *Haemophilus* and *Neisseria* were higher in NE group when compared to C group. *Abiotrophia* and *Oribacterium* were higher in OLP (combining E and NE groups), while *Eikenella* and *Aggregatibacter* were lower when compared to C group. There was statistically significance in α-diversity between E and RAU groups(*p* < 0.05). Significant differences in β-diversity were detected in bacteria between E and C; NE and C; as well as E and NE groups. The LDA effect size algorithm identified the *g_Haemophilus* might be the potential biomarker in NE group.

**Conclusions:**

We found that salivary microbiome in erosive OLP was significantly different from that found in RAU; and these changes may be related to the underlying disease process rather than presence of ulcerative/erosive lesions clinically. In addition, our findings in bacterial relative abundance in OLP were significantly different from the previously reported findings, which points to the need for further research in salivary microbiome of OLP.

## Background

Oral lichen planus (OLP) is a common oral mucosal disease with or without accompanying lesions in skin, nails, eyes, or urogenital tissue [1]. OLP is regarded as a chronic T-cell-mediated inflammatory disease, exhibiting a higher prevalence in women than men, especially those over fifty years of age [2, 3]. The lesions typically spread throughout the oral mucosa and usually involve the posterior buccal mucosa bilaterally [3]. There are essentially two forms of oral lesions in OLP: erosive, and reticular or non-erosive [4], with the latter being the most common. However, erosive lesions are more significant to the patients because they usually cause sore mouth and may affect the ability to eat and maintain good oral hygiene [5]. Notably, it is common for patients to have both reticular and erosive lesions. Although the etiology of OLP is still unknown, various factors have been suggested to contribute to the pathogenesis, including systemic medications [6, 7], dental restorative materials such as amalgam [8] and composite or resin-based materials [9], cinnamon-containing products, and microorganisms.

Whether oral microbial changes in OLP disease state is not clear, but some studies have confirmed the correlation between OLP and oral microorganism [10]. Bornstein et al. [5] investigated microbial in OLP patients with non-erosive/asymptomatic lesions and found that bacterial counts for *Capnocytophaga sputigena*, *Eikenella corrodens*, *Lactobacillus crispatus*, *Mobiluncus curtisii*, *Neisseria mucosa*, *Prevotella bivia*, *Prevotella intermedia*, and *S. agalactiae* at the sites of OLP lesions are significantly higher when compared to the same sites in control subjects. In addition, bacterial counts for *Bacteroides ureolyticus*, *Dialister* species, *Staphylococcus haemolyticus*, and *Streptococcus agalactiae* were also significantly higher in OLP gingival lesions when compared to those found in the contralateral unaffected sites within the same patient. Since these OLP patients were asymptomatic, the differences in microbial species and quantity found in OLP lesions are most likely due to OLP disease process but not patients’ inadequate hygiene maintenance caused by soreness/discomfort from these OLP lesions. Although the clinical types (erosive or reticular) and presence or absence of symptoms were not specified, Ertugrul et al. [11] also found that the proportion of *Aggregatibacter actinomycetemcomitans*, *Porphyromonas gingivalis*, *Prevotella intermedia*, *Tannerella forsythia*, and *Treponema denticola* in total bacteria were higher in subgingival plaque samples taken from subjects having periodontitis and OLP than those having periodontitis without OLP. The results of these studies suggest that there may be correlation between the microbe and OLP. To the best of our knowledge, there has been only two studies investigating salivary microbiome in OLP in the literature so far [12, 13], and the changes of oral microbiome in OLP remains unclear.

Therefore, the purpose of this study was to identify the characteristic microbial profile in the saliva of OLP patients with or without erosive lesion. The difference between erosive OLP and non-erosive OLP is the presence of multiple ulcerative lesions in the clinical presentation of OLP. It is likely that the presence of oral ulcerative lesions may have affected oral microbiome distribution already, without an underlying mucocutaneous disease. Therefore, a group of recurrent aphthous ulceration (RAU) patients was also included as a comparison to further test if the microbiota changes occurred in OLP would be unique to OLP. RAU is a common oral mucosal disease also characterized by multiple oral ulcers, a feature similar to erosive OLP. Both OLP and RAU are T cell-mediated immunological disorders; however, the pattern of the clinical lesions and the pathogenesis mechanism of RAU are different from OLP except the common clinical feature of multiple ulcerations [14]. Using the Illumina MiSeq sequencing, a high-throughput sequencing technology, we examined the relative abundances in different bacteria, the diversity of bacteria, and the dominant bacteria species in different study groups. The results reveal a distinctive bacterial signature for OLP, which is different from RAU, and shed a light to the different underlying immunological mechanism of these two diseases.

## Results

### Characterization of study subjects and microbial analysis

The demographic and clinical characteristics of the four study groups: OLP patients with erosive lesions (E), OLP patients without erosive lesions (NE), patients with oral aphthous ulcer (U) and healthy controls (C) were shown in Table 1. All subjects were free of hypertension, diabetes, smoking cigarette habit and alcohol-drinking. By t-test, there was no significant differences in age, gender, and saliva pH, respectively, among groups (*p* < 0.05).

**Table 1:** Patient Demographics, Clinical History and Salivary pH

Characteristic	Non-erosive LP (***n*** = 10)	Erosive LP (***n*** = 10)	Ulcer (***n*** = 10)	Healthy Controls (***n*** = 10)
Age(mean ± SD)	46.6 ± 12.1	51.6 ± 14.2	34.7 ± 17.3	47.0 ± 13.27
Male/Female	3/7	4/6	4/6	6/4
Smoking cigarettes	0	0	0	0
Drinking alcohol	0	0	0	0
Hypertension	0	0	0	0
Diabetes	0	0	0	0
Salivary pH (mean ± SD)	6.45 ± 0.50	6.20 ± 0.42	6.50 ± 0.48	6.60 ± 0.52

A total of 1,173,674 clean reads were obtained from the analysis of saliva collected from all subjects. An average of 9236 high quality sequences between 200 and 400 bp in length were clustered into 4949 operational taxonomic units (OTUs) with a 97% identity of coverage for each group.

### Alpha diversity of microbiome in each group

**Fig. 1:**
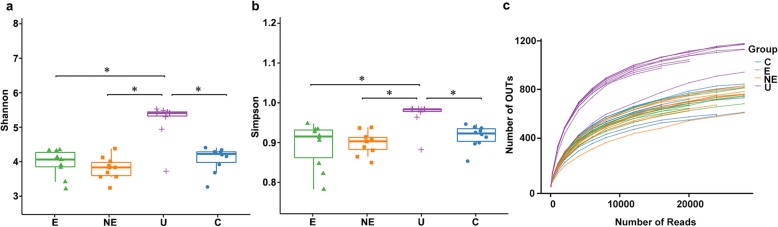
Alpha diversity measurement in saliva from patients with erosive (E), non-erosive (NE), aphthous ulcer (U) and control (C). **a** Simpson index of diversity, **b** Shannon index of diversity, and **c** Rarefaction curve plot. (*: *p* < 0.05)

The salivary bacterial community in E, NE, U and C groups was first analyzed quantitatively by Shannon and Simpson diversity indices, respectively. No significant differences were observed in the comparison between NE and C or E and C (Fig. 1a and b). The index value of the U group was significantly higher than those found in any of the other three groups.

Samples from each group were further analyzed by rarefaction curves, which showed that the number of OTUs was also higher in the saliva samples from U group than those found in NE, E or C groups, respectively (Fig. 1c).

The rarefaction curves indicated that almost entire bacterial population in our samples of OLP saliva was represented in our results based on 97% good’s coverage. However, the present curves showed sufficient sequencing depth, if we added more saliva samples, number of OTUs might concomitantly increase. Next, we used the 16 s rDNA gene amplicon sequencing to perform detailed analyses of the relative abundance, variation of bacteria and dominant bacteria.

### Relative abundance of microbiota in each group

**Fig. 2:**
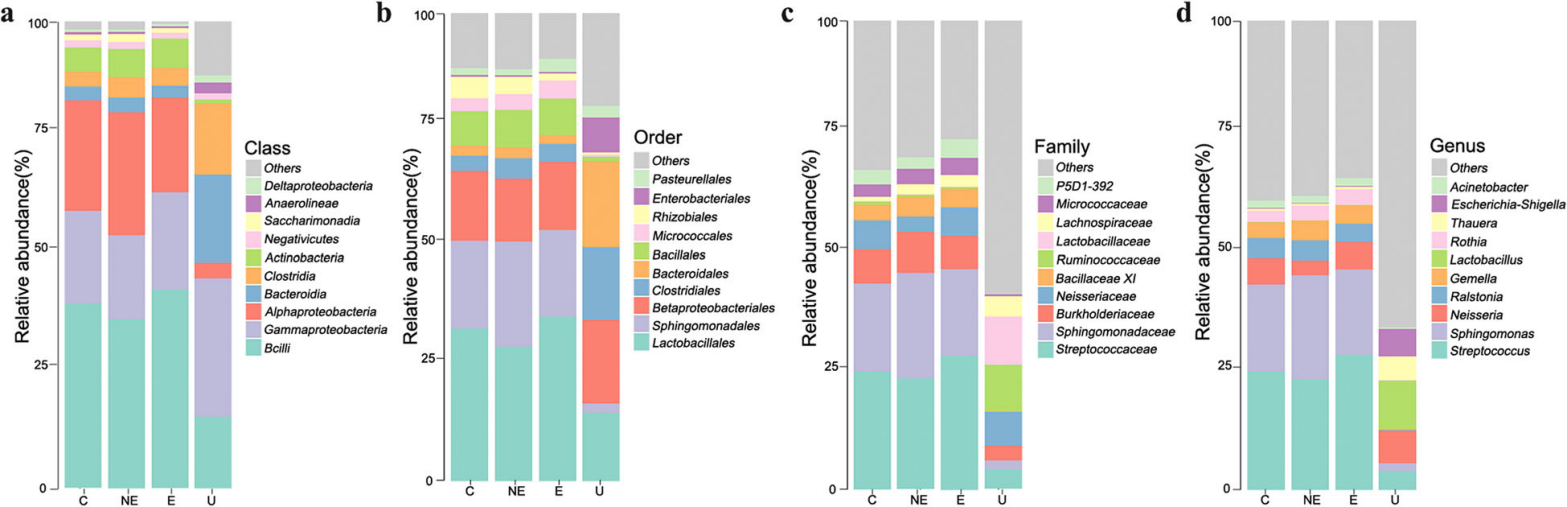
Relative abundance of oral microbiota at different taxomal levels and comparison among control (C), non-erosive (NE) and erosive (E) OLP, with (a to d) aphthous ulcer (U) group. **a**-**d** Representation of the mean relative abundance at class, order, family, and genera level respectively in four groups. **c** control group; **e** erosive OLP group; NE: non-erosive OLP group; U: recurrent aphthous ulcer)

To further explore the evenness and divergence of bacterial community in the saliva of each group, we analyzed the relative abundance of microbiome at different taxomal levels (Fig. 2). While NE, E and C groups showed similar pattern of dominant bacteria, the U group showed significant differences from the other three groups at all (class, order, family and genus) levels (2 a to d). At the class level, bacilli, the relative abundance of *Alphaproteobacteria*, *Actinobacteria*, and *Saccharimonadia* were less, but *Gammaproteobacteria*, *Bacteroidia*, and *Clostridia* were more in the U group when compared with those found in the other three groups (2a). At the order level, *Lactobacillales*, *Sphigomonadales*, *Bacillales* and *Micrococcales* were less abundant, but *Clostridiales*, *Bacteroidales*, and *Enterobacteriales* were more abundant when compared with those found in the other three groups (2b). At the family level, *Streptococcaceae*, *Sphingomonadacae*, *Burkholderlaceae*, *Micrococcaceae* and P5D1–392 were less abundant, but *Lactobacillaceae* and *Ruminococcaceae* were more abundant in the saliva of the U group when compared with those found in the other three groups (2c). At genus level, *Streptococcus* and *Sphingomonas* were two of the most abundant bacteria species found in the NE, E and C groups, while they were two of the least abundant bacteria species found in the U group. In addition, the relative abundance of *Lactobaccilus*, *Escherichia-Shigella*, and *Thauera* were much more in the saliva of the U group when compared with those found in the other three groups (2d).

### Beta diversity of microbiome between OLP and healthy control

Since the major purpose of our study was to investigate difference in salivary microbiome in OLP from that in the healthy controls, we therefore focused on the investigation of the OTU-based beta diversity of microbial community in OLP patients’ saliva and compared that with the control.

Significant differences were observed in the comparison of samples between E and C, NE and C, as well as E and NE groups, respectively, when the beta diversity of each group were analyzed by phylogenetic unweighted UniFrac-based method (*p* < 0.005, Table 2), and Jaccard-based analysis considering only presence-absence of sequences in a community (*p* < 0.005, Table 2). This suggested the microbial diversity among samples in the same group was significantly different from each other.

**Table 2:** Comparison of Beta Diversity Among Three Study Groups Using Unweighted UniFrac and Jaccard Analysis. (Number represents the *p*-value; E, NE and C represent erosive-OLP, non-erosive OLP and controls, respectively)

Group	unwei_unifrac	Jaccard
NE vs. C	0.001	0.019
E vs. C	0.003	0.042
E vs. NE	0.001	0.05
E vs. NE vs. C	0.001	0.005

**Fig. 3:**
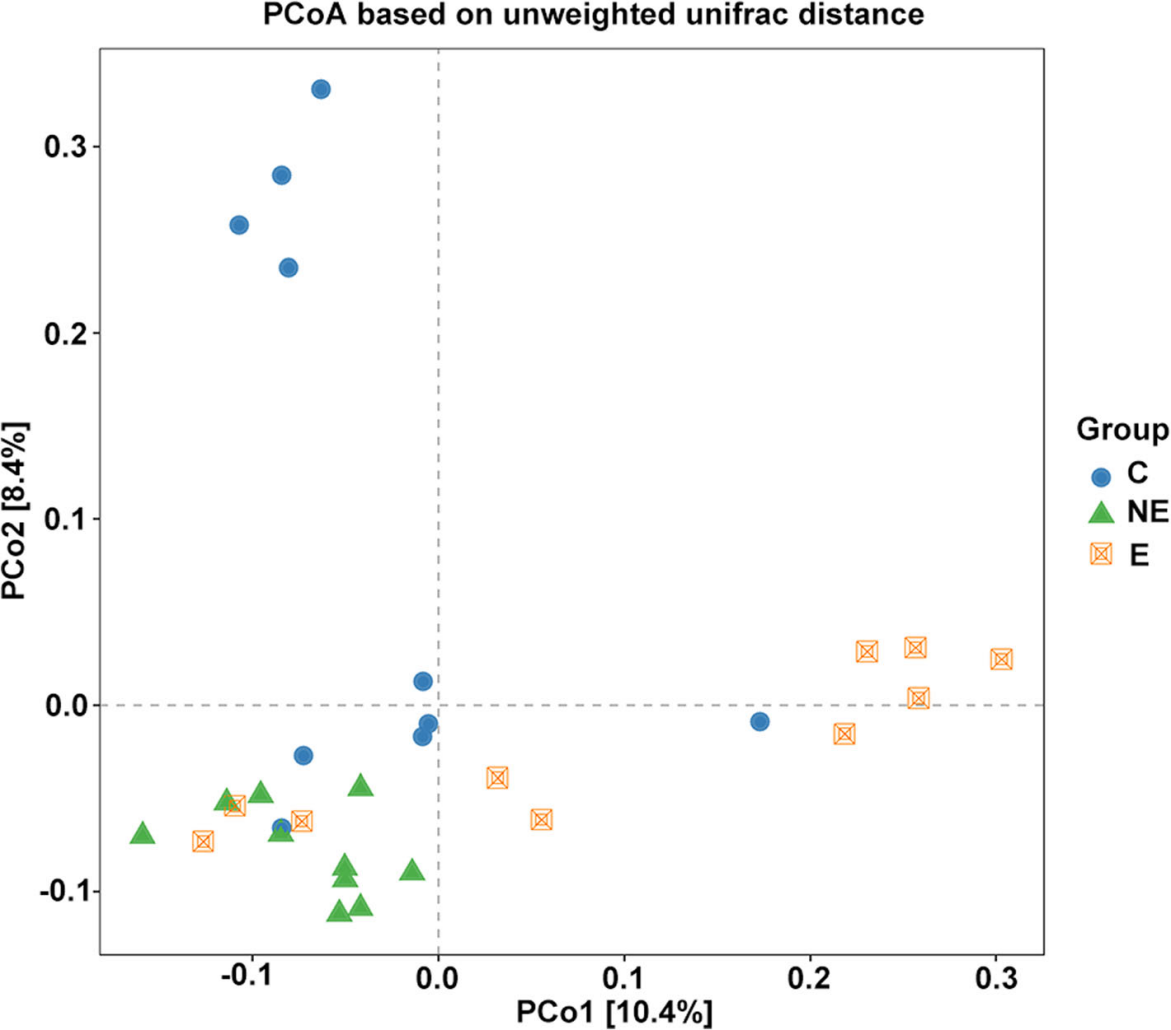
Beta diversity analysis. The microbial community compositional differences between each group; controls, erosive OLP, and non-erosive OLP groups. PCoA plot based on the unweighted UniFrac distance matrix showing the divergence in distribution of the oral microbiota from different samples/group. PC1, and PC2 labeled on the X and Y axis represent the top two principal coordinates that captured the fraction of diversity at the coordinate shown percent (NE: green triangle; E: orange double square; and C: blue circle). The distribution of samples in NE showed a cluster, which was distinguishable from samples in C group and the majority of samples in E group. The distribution of samples in E and in C groups appeared to be expanded broadly toward the direction of right of PCo1 and up of PCo2, respectively

The comparison of beta diversity relationships in the saliva from each group was plotted by a Principle coordinate analysis (PCoA) based on the methods of unweighted UniFrac distance (Fig. 3). The distance between marks represents how different compositionally the samples were from each other within the same group or among different groups (with different marks). In Fig. 3, the distribution of principal coordinate 1 (PCo1) accounts for 10.4% of the variance in data and PCo2 accounts for 8.4% of the variance. The distribution of samples in NE showed a cluster, which was distinguishable from samples in C group and the majority of samples in E group (Fig. 3). The bacterial diversity of each sample in the NE group appeared to be more closely related or similar (divergence-based measures), and was different from those found in the samples in C and in E groups, while the OTUs in the samples of E or C group was less related and more heterogeneous.

### Bacterial community of each group and core microbiome

**Fig. 4:**
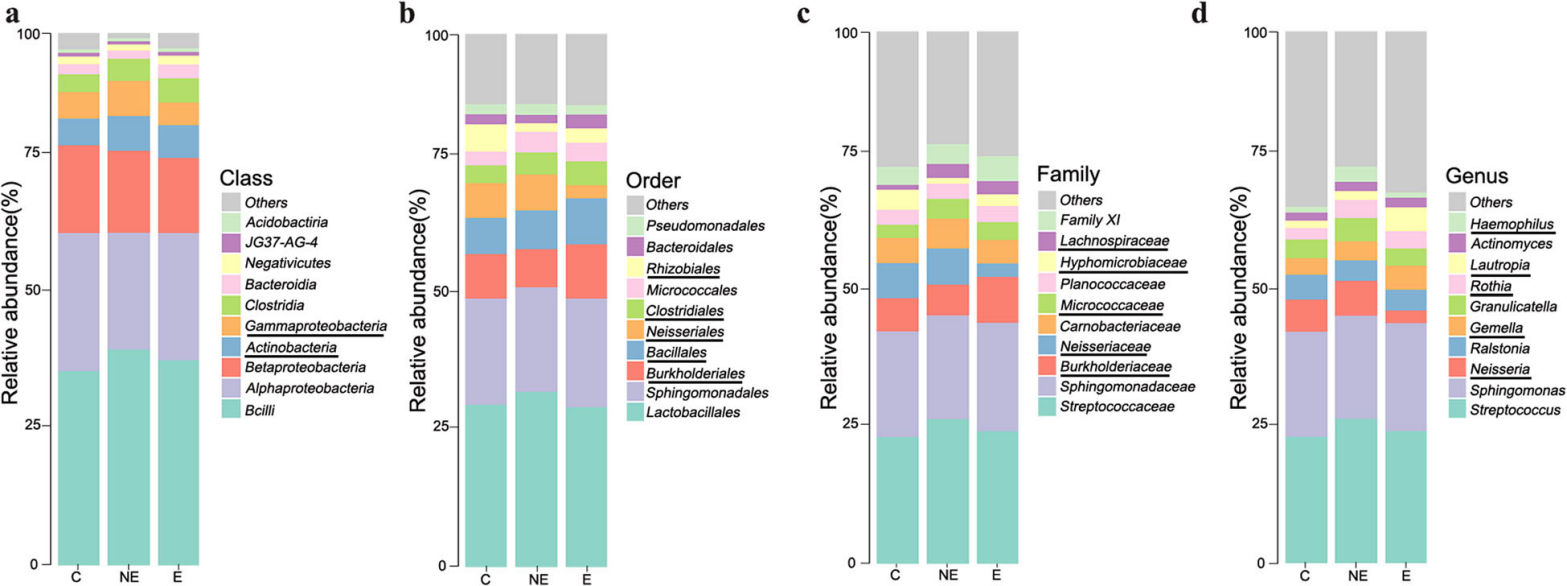
Relative abundance of oral microbiota at different taxomal levels and comparison among control (C), non-erosive (NE) and erosive (E) OLP. **a**-**d** Representation of the mean relative abundance at class, order, family, and genera level, respectively. The underline of bacteria represents the increased or decreased relative abundance of oral bacteria in OLP disease group. **c** control group; **e** erosive OLP group; NE: non-erosive OLP group)

We further examined the relative abundance of bacterial communities in the saliva of the NE and E groups, and compared them to those found in the saliva of the C group. Among the bacterial communities in each groups, dominant bacteria were identified at different taxomal levels in the saliva of these three groups’ (Fig. 4 and 4S).

The relative abundance of the following bacteria in both OLP groups (E and NE) were increased when compared to the healthy control; *Actinobacteria* and *Clostridia* (at class level; 4a), *Bacillales* and *Clostridiales* (at order level; 4b), *Lachnospiraceae* and *Micrococcaceae* (at family level, 4c), and *Rothia*, *Gemella*, as well as *Lautropia* (at genus level; 4d), whereas the relative abundance of the following bacterial species in the OLP groups were decreased; *Betaproteobacteria* (at class level; 2a), *Rhizobiales* (at order level; 2b), and *Hyphomicrobiaceae* (at family level; 2c). However, these findings were not statistically significant with the Wilcoxon test. Notably, at the genus level, *Lautropia* in the saliva of E group was much more abundant than it was in either NE or C groups. In contrast, *Neisseriales*, *Neisseriaceae*, and *Neisseria* at the level of order, family and genus, respectively, in the saliva from E group showed dramatically less abundant than they were in either C or NE groups (Fig. 4)*.*

**Fig. 5:**
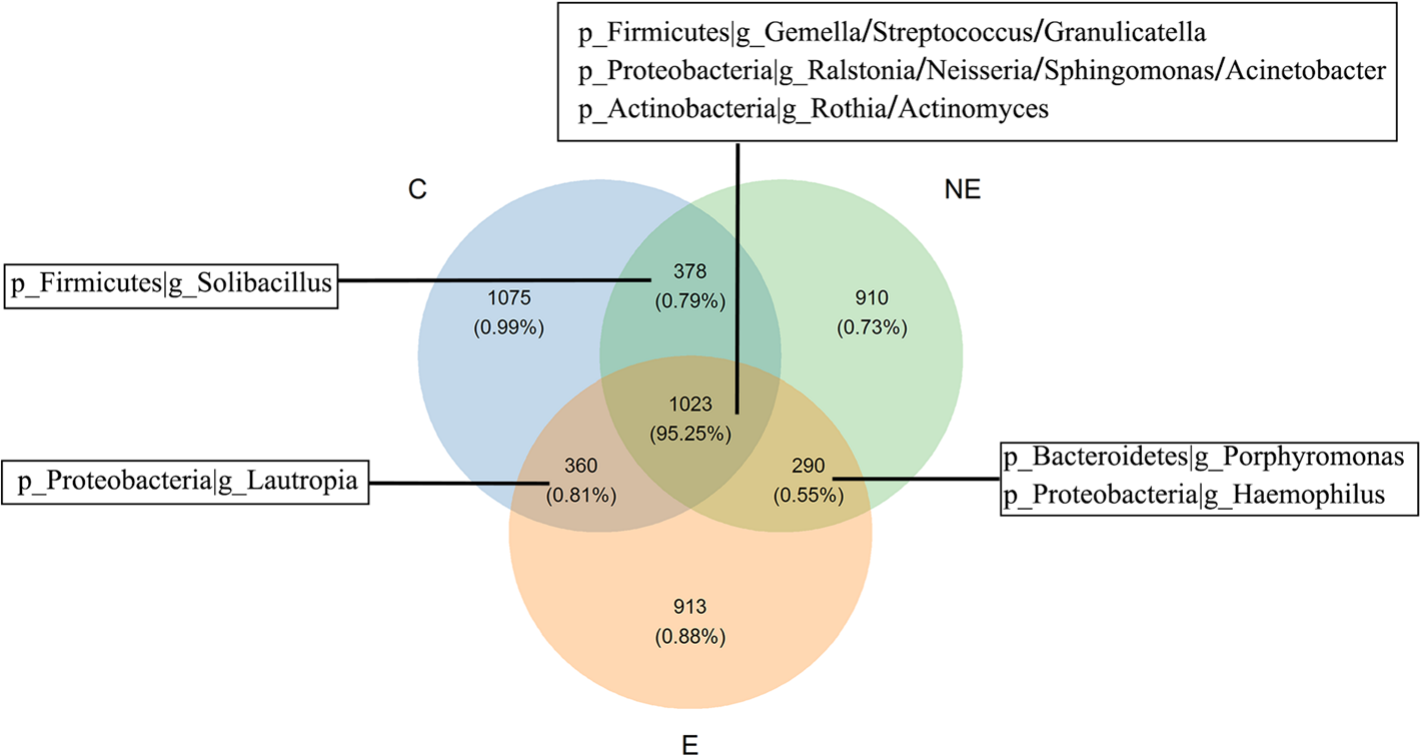
Venn diagram shows core microbiome among the NE, E and C groups based on OUT analysis. The co-occurrence in OTU number is shown along with the overlap of the microbiota at genus level in three groups. The number represent OTUs being sequenced, and the percentages below represent the proportion of corresponding OTUs divided by total OTUs sequenced in the analysis

The core microbiome analysis based on OTUs in the saliva from these three groups was visualized by Venn figure analysis (Fig. 5). The core site contained 1023 OTUs, counting for 95.25% of total sequencing OTUs shared by the three groups*.* The bacterial membership presented in the core site included the top 9 dominant genus under three phylum in the abundance analysis (Fig. 4S); *Firmicutes* (*g_Gemella, Streptococcus*, and *Granulicatella*), *Proteobacteria* (*g_Ralstonia*, *Neisseria*, *Acinetobacter*, and *Sphingomonas*) *and Actinobacteria* (*g_Rothia* and *Actinomyces*). The association among these core microbiome was further analyzed by correlation coefficient (Table 3) to exam their possible interaction, that was, whether one presence was related to the other. The results showed that the presence and richness of *Streptococaceae* was negatively correlated with *Sphingomonadaceae*, and *Ralstonia* (*p* < 0.001 and *p* < 0.001, respectively, Table 3), which indicated the increase of *Streptococaceae* in a group was associated with the decrease of *Sphingomonadaceae*, and *Ralstonia*, respectively, in the same group. Negative correlation was also observed between *Sphingomonas* VS *Gemella* and *Ralstonia* VS *Gemella* (*p* < 0.001 and *p* < 0.001, respectively, Table 3). Nevertheless, the presence of *Gemella* VS *Streptococcus* and *Sphingomonas* VS *Ralstonia* were positively correlated (*p* < 0.001 and *p* < 0.001, respectively, Table 3), suggested that the increase of *Gemella* and *Sphinogomonas* were correlated with the increase of *Streptococcus* and *Ralstonia*, respectively.

**Table 3:** The correlation coefficient between bacteria in total simples

Bacteria component	R	***p***. value
*Streptococcus* VS *Sphingomonas*	−0.780	< 0.001
*Streptococcus* VS *Neisseria*	−0.101	0.593
*Streptococcus* VS *Ralstonia*	−0.789	< 0.001
*Streptococcus* VS *Gemella*	0.869	< 0.001
*Streptococcus* VS *Actinomyces*	0.392	< 0.05
*Streptococcus* VS *Haemophilus*	0.505	0.127
*Sphingomonas* VS *Neisseria*	−0.080	0.672
*Sphingomonas* VS *Gemella*	−0.790	< 0.001
*Neisseria* VS *Actinomyce*	0.332	0.072
*Sphingomonas* VS *Ralstonia*	0.889	< 0.001
*Neisseria* VS *Ralstonia*	−0.028	0.882
*Ralstonia* VS *Gemella*	−0.775	< 0.001

R: Spearman rank correlation coefficient

On the other hand, the overlap area between E and NE groups in Fig. 5 containing 290 OTUs were “variable” bacteria: *g_Porphyromonas* and *g_Haemophilus*. While the overlap OTUs between C and NE was identified as *g_Solibacillus*, the overlap OTUs between C and E was *g_Lautropia*. Correlation coefficient analysis did not reveal that the changes of *Neisseria and Haemophilus* in a group were associated with the richness of any identified dominant bacteria in the core site (Table 3). This indicated that the changes in richness of the two bacteria: *g_ Neisseria* and *g_Haemophilus* might be unique to the occurrence of OLP*.*

### Differential microbial composition in each group

**Fig. 6:**
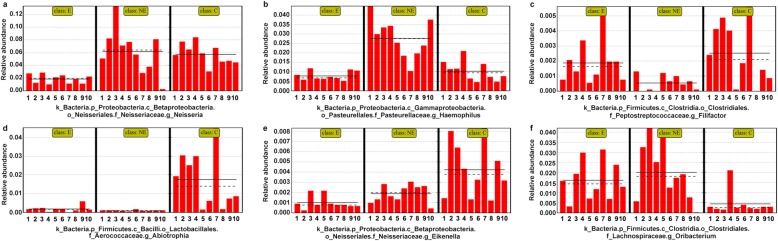
Summary of the differential bacteria that can distinguish OLP from the healthy controls. **a**-**f** Data was based on the results from LEfSe method. The red bar graph displayed that the changing of dominant biomarkers in different groups

In search for salivary microbiome distinguished OLP from the health control, linear discriminant analysis (LDA) effect size (LEfSe) was used for the quantitative analysis within different groups [15]. We illustrated the potential dominant microbiome that could distinguish OLP (NE and E) from the healthy controls (C). It showed that *g_Abiotrophia* (*f_Aerococcaceae*) and *g_Eikenella* were dramatically decreased, while genera *Oribacterium* (*f_Lachnospiraceae*) was increased significantly in the OLP groups when compared to those found in the health controls (Fig. 6d, e and f). As far as the specific bacteria found in the saliva samples of the OLP patients, *g_Haemophilus* (*c_Gammaproteobacteria*, *o_Pasteurellales*, *f_Pasteurellaceae*) was significantly increased (Fig. 6b) in the NE group, whereas genera *Neisseria* (*o_Neisseriales* and *f_Neisseriaceae*) was dramatically decreased in the E group (Fig. 6a). Therefore, the *g_Haemophilus* might be the potential biomarker in NE group.

**Fig. 7:**
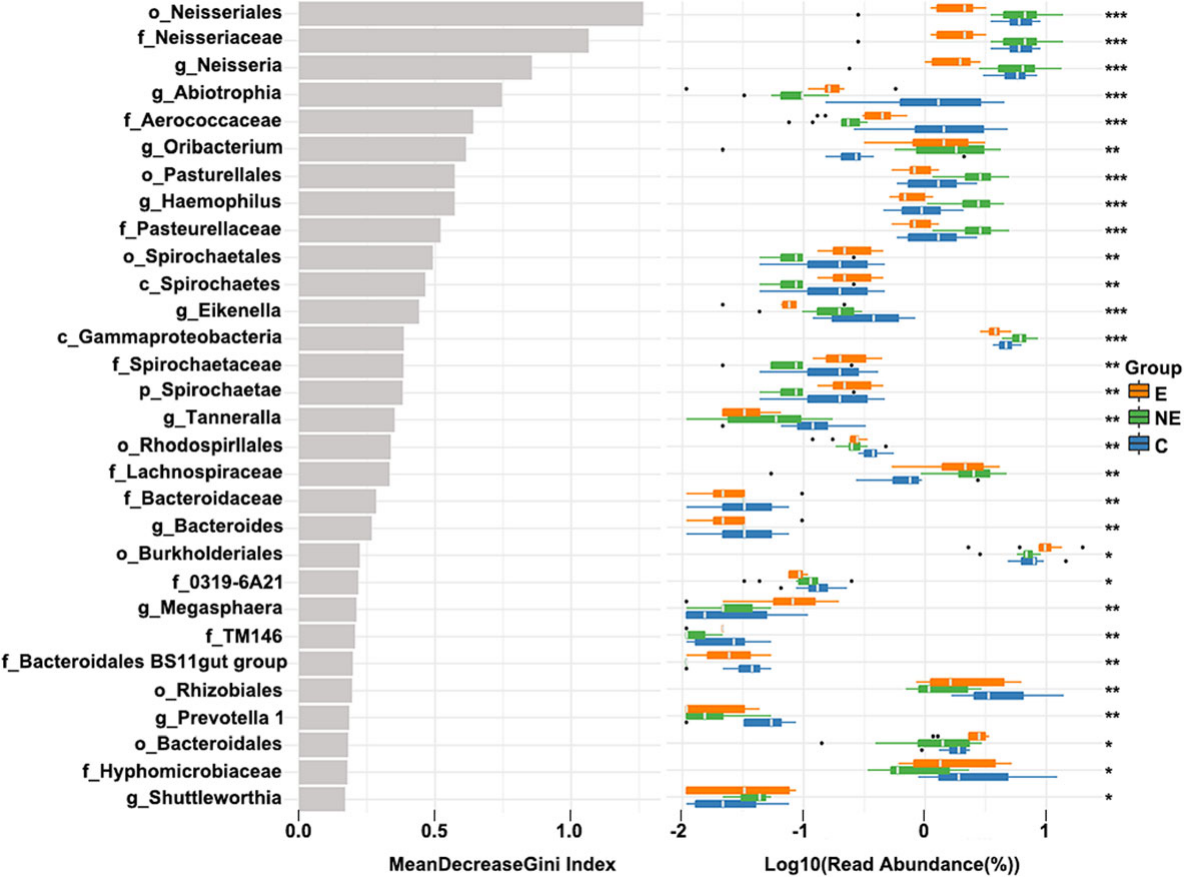
The Mean Decrease Gini Index in E, NE and C groups. The abscissa on the left figure was the mean decrease Gini index, and the ordinate represented the bacteria classification. The right box plot was the relative abundance bacteria in three groups (***: *p* < 0.001; **: *p* < 0.01; *: *p* < 0.05)

The Mean Decrease Gini Index of Random Forest analysis was also performed to investigate the different important microbial species among the groups (Fig. 7, Table 4). It showed that bacteria *g_Abiotrophia* and *f_Aerococcaceae* were significantly decreased, whereas *g_Oribacterium* was significantly increased in the saliva samples of the E and NE OLP groups when compared to those found in the saliva samples of the health controls (*p* < 0.01, Table 4). The bacteria *g_Neisseria* was significantly reduced in the saliva samples of the E group when compared to that found in the saliva samples of either NE or C groups (*p* < 0.01, Table 4). While *g_Haemophilus* was significantly increased, *g_Spirochaetes* and *o_Spirochaetales* were significantly reduced in the saliva samples of the NE group (*p* < 0.01, Table 4).

**Table 4:** The differential Taxa of Bacteria Among Three Study Groups

Taxa	Erosive OLP	Non-erosive OLP	Control	***p***. value
*g_Neisseria*	1.85 ± 0.71	6.19 ± 3.60	5.75 ± 1.65	< 0.001
*g_Abiotrophia*	0.18 ± 0.16	0.09 ± 0.04	1.75 ± 1.48	< 0.001
*g_Haemophilus*	0.78 ± 0.24	2.76 ± 1.02	1.03 ± 0.53	< 0.001
*g_Spirochaetes*	0.26 ± 0.12	0.08 ± 0.07	0.22 ± 0.15	< 0.001
*g_Oribacterium*	1.61 ± 0.97	1.99 ± 1.41	0.44 ± 0.59	< 0.01
*f_Aerococcaceae*	0.42 ± 0.19	0.23 ± 0.08	1.93 ± 1.51	< 0.001
*o_Spirochaetales*	0.27 ± 0.12	0.08 ± 0.06	0.22 ± 0.15	< 0.01

Data was presented as mean ± SD, which was the log value from the Mean Decrease Gini index of each group, developed from the operational taxonomic unit (OTU)-level abundances (read abundance %) analyzed by Random Forest classification. The higher the value, the less read abundance the bacteria

When we compared OLP group, by combining NE and E groups, with the healthy control, we found that *g_Oribacterium* level was significantly higher whereas the levels of *g_Abiotrophia* and *g_Oribacterium*, *g_Eikenella*, *g_Aggregatibacter*, *g_Bacteroides*, *g_Neisseria*, and *g_Ezakiella* were all significantly lower in OLP groups than those found in the healthy control group (Fig. 7S and Table 5).

**Table 5:** The Differential Taxa of Bacteria Between OLP (combined E and NE) and Control Groups

Taxa	OLP	Control	***p***. value
*g_Oribacterium*	1.81 ± 1.22	0.44 ± 0.59	< 0.001
*g_Abiotrophia*	0.13 ± 0.10	1.75 ± 1.48	< 0.001
*g_Eikenella*	0.12 ± 0.10	0.34 ± 0.26	< 0.01
*g_Aggregatibacter*	0.11 ± 0.10	0.22 ± 0.15	< 0.01
*g_Bacteroides*	0.02 ± 0.03	0.04 ± 0.03	< 0.01
*g_Neisseria*	4.10 ± 3.50	5.75 ± 1.65	< 0.05
*g_Ezakiella*	0	0.11 ± 0.05	< 0.001

Data was presented as mean ± SD, which was the log value from the Mean Decrease Gini index of OLP and control group, the method was similar to Table 4

## Discussion

In the present study, we investigated the complex microbial community in the saliva of OLP patients with or without erosive lesions, group E and group NE, respectively; and compared that found in RAU. Our results in alpha diversity (Fig. 1) showed that there was no significant difference in bacterial species between these two OLP groups (E and NE). In addition, the bacterial species in OLP, including both E and NE groups, were significantly less diversified than those found in the RAU (group U). The relative abundance of oral microbiota (Fig. 2) also showed that the dominant bacteria in OLP, including both E and NE, was significantly different from those found in the U group at all levels (class, order, family and genus). These results demonstrated that even though multiple ulcerations are seen in both OLP and RAU, the salivary microbiome in these two disease processes are significantly different. These results suggest that the salivary microbiome may correlate directly with the underlying disease process but not presence of oral ulcerations.

Whether the salivary oral microbiome changes in OLP and RAU seen in our study are the *result* or the *cause* of these two different immunological disorders is unknown. The etiology for both OLP and RAU are currently unclear; however, microorganism has been implied as one of the potential causes for both disorders. The etiological factors of OLP include hypersensitivity due to local or systemic inducers, autoimmune, psychological stress and micro-organisms (especially hepatitis C virus) [16]. The etiological factors of RAU include genetic predisposition (HLA-B12, B51 and Cw7), nutritional deficiencies, immunologic diseases (cyclic neutropenia, acquired immunodeficiency syndrome), hormonal influences, microorganism (bacteria), trauma, stress and food allergies [17]. The results of our study unfortunately cannot provide evidence to indicate whether microbiome differences are the cause or the result of a disease process. Nevertheless, we found that *Streptococcus* and *Sphingomonas* were two of the most abundant bacteria species found in saliva of the OLP patients but they were two of the least abundant bacteria species found in RAU (Fig. 2). In addition, the relative abundance of *Lactobaccilus*, *Escherichia-Shigella*, and *Thauera* were much more in saliva from RAU patients when compared with those found in OLP patients. These findings may point to a direction for future research effort in the correspondent host immunologic responses and possible cause-effect relationships of these two distinctive immunological disorders.

Latest researches systematically reviewed the microorganisms might be responsible for OLP, with differences in results from buccal mucosa and saliva specimens [10, 12, 13, 18, 19]. In here, we listed our results with those previously reported by Wang et al. and other three researchers [12, 13, 18, 19] in Table 6. In this study, we found that several bacterial species appeared to show significantly different abundance in OLP when compared to healthy controls (Table 5). The level of *g_Oribacterium* was significantly higher in OLP whereas the levels of *g_Abiotrophia*, *g_Eikenella*, *g_Aggregatibacter*, *g_Bacteroides*, *f_Neisseria*, and *o_Ezakiella* were all significantly lower in OLP than those found in the healthy control group (Table 5). Interestingly, the above-mentioned bacterial species did not significant difference in their abundance in Wang’s and Li’s study [12, 13]. In contrast, those found to be significantly higher or lower in their relative abundance in Wang’s study, such as *Porphyromonas*, *Solobacterium*, *Corynebacterium*, *Cellulosimicrobium*, *Campylobacter* did not show significant difference in their abundance in our OLP patients population. The two bacteria species that showed significantly lower relative abundance in OLP patients in Wang’s study, *Haemophilus* and *Rothia aeria*, in fact showed significantly higher relative abundance in our study (Table 6). The reason for these differences in findings is unknown. Li et al. found *Aspergillus* and *Candida* higher abundance in reticular OLP patients, while *Alternaria* and *Sclerotiniaceae_unidentified* increased in erosive OLP group. Meanwhile, they demonstrated several fungal were significant correlation with IL-17 and clinical scores. Therefore, we would supplement the correlation between high abundant bacteria and inflammatory factors on OLP and further to provide clinical guidance for this diseases. He et al. and Du et al. [18, 19] found *Fusobacterium* increased in OLP group compared to healthy control in buccal mucosa. Their results were different from ours which might be caused by different types of samples. Wang’s and Li’s patient population were in Southern China, while ours was in Northern China. It is known that common food and dietary habits are different in Southern from Northern China and we speculate that perhaps this may play a role in the differences in our findings.

**Table 6:** Comparison of the Results Found in other Researchers and the Present Study Regarding Higher Abundant Salivary Bacteria Species in Different Groups. (No: no co-bacteria)

Researchers	Sample	Groups	Taxa of Bacteria	Co-bacteria
Wang et al. (2016)	salivary	Erosive OLP group	*Porphyromonas/ Veillonella parvula*	No
		Non-erosive OLP group	*Solobacterium/Actinomyces* *Prevotella melaninogenica/ Veillonella parvula*	No
		Healthy group	*Haemophilus*/*Corynebacterium/* *Cellulosimicrobium/Campylobacter/*	No
He et al. (2017)	buccal mucosa	OLP group (erosive/non-erosive)	*Fusobacterium/Leptotrichia/* *Lautropia*	No
		Healthy group	*Streptococcus*	No
Du et al. (2019)	buccal mucosa	Erosive OLP group	*Fusobacterium/ Granulicatella/* *Prevotella/ Bacillus/F. nucleatum*	No
		Reticular OLP group		No
		Healthy group	*Streptococcus/ Neisseria*	*Neisseria*
Li et al. (2019)	salivary	Erosive OLP group	*Alternaria/Sclerotiniaceae_unidentified*	No
		Reticular OLP group	*Aspergillus/ Candida*	No
		Healthy group	*Ascomycota_unidentified_1_1/Trichosporon*	No
Present results	salivary	Erosive OLP group	*Rothia / Oribacterium*	–
		Non-erosive OLP group	*Haemophilus/ Oribacterium*	
		Healthy group	*Abiotrophia/ Eikenella/ Aggregatibacter/ Bacteroides/ Neisseria/ Ezakiella*	
		RAU group	*Lactobaccilus/Escherichia/Shigella*/*Thauera*	

An interesting finding in the beta diversity of microbiome in OLP was that the microbial community among different patients’ saliva samples in the NE group appeared to be more consistent and showed less variety, whereas microbiome in the saliva samples in E and in C groups appeared to be more variable and diverse (Fig. 3). It may indicate a unique microbiome pattern that may distinguish non-erosive OLP from erosive OLP and the healthy control. However, it is the limitation of our study that the sample size for each group is relatively small (*n* = 10). A large-scale study is need to confirm this preliminary interesting finding.

## Conclusions

We investigated salivary microbiome in OLP and compared that with RAU and healthy controls. We found that microbiome in erosive OLP was significantly different from that found in RAU; and microbiome changes may be related to the underlying disease process rather than presence of ulcerative/erosive lesions clinically. In addition, our findings in bacterial relative abundance in OLP were significantly different from the previously reported findings, which indicate further research in salivary microbiome in OLP.

## Methods

### Ethics statement and subject recruitment

The study was approved by the Ethics Committee of the School of Stomatology, Shanxi Medical University (No.2017LL018). Samples were collection after receiving the consent. The inclusion criteria were patients who were diagnosed with OLP or RAU by the pathologists of the Department of Pathology, School of Stomatology, Shanxi Medical University. All OLP patients had biopsies confirming their diagnoses. In addition, all participants met the following conditions:

1) did not take any immunosuppressive drugs 3 months prior to saliva collection; 2) had a teeth cleaning at least 3 months prior to saliva collection; and 3) did not take any food at least 2 h before sample collection in morning. Depending on the clinical presentation, patients with OLP were divided into two groups: erosive OLP (n = 10, group E) and non-erosive OLP (n = 10, group NE). Ten healthy individuals were recruited as healthy controls (group C); and ten patients with RAU (group U) were recruited as a comparison (see reasons stated in the Introduction).

### Sample collection and processing

Whole saliva was collected from participants between 8 am and 12 pm, according to previously published protocol [20]. All participants were normally performed clinical examination before collecting saliva sample, and prevented from eating and drinking. The participants were asked to sit upright and approximately 2 ml of un-stimulated whole saliva were collected. Saliva samples were kept in ice during collection, and transported to the laboratory within 2 h. The samples were then centrifuged at 2600 g at 4 °C for 20 min and stored at − 80 °C until further use [21].

### Extraction of genomic DNA and PCR amplification

Total genomic DNA in the salivary sample was extracted by the DNeasy PowerSoil Kit (QIAGEN, Hilden, Germany) according to manufacturer’s protocol. The concentration and quality of each extracted DNA sample was determined by NanoDrop Spectrophotometer (Thermo Scientific, Waltham, MA, USA). Each DNA sample was further diluted to 10 ng/μl with sterile ultrapure water, and stored at − 80 °C before analysis. Two PCR primers with 12 nt unique barcode; the forward primer 515F (5′-GTG YCA GCM GCC GCG GTA A-3′) and reverse primer 806R (5′-GGA CTA CHV GGG TWT CTA AT-3′) were used to amplify the V4 hypervariable 16S rRNA genes [22]. Fifty microliter PCR mixture was prepared for the PCR amplification, which contained 2× PCR buffer with 1.0 μM forward and reverse of each primers, 1.5 mM MgCl_2_, 0.4 μM each deoxynucleoside triphosphate, 0.5 U DNA polymerase (KOD-Plus-Neo, Toyobo), and 10 ng template DNA. The PCR amplification was conducted by an initial denaturation at 94 °C for 1 min, followed by 30 cycles of denaturation at 94 °C for 20 s, annealing at 55 °C for 30 s, and elongation at 72 °C for 30 s, and a final extension at 72 °C for 5 min. Three replicates of PCR products for each sample were pooled together, which were then loaded on 2% agarose gels for the detection of the primary band between 200 and 400 bp. The electrophoresis band was cut off and the DNA products in the gel was extracted using an OMEGA Gel Extraction Kit (Omega Bio-Tek), followed by quantification (by Qubit@ 2.0 Fluorometer, Thermo Scientific). The PCR products from different samples were pooled with equal molar amounts for paired-end sequencing.

### 16 s rDNA gene library sequencing and processing of sequencing data

The sequencing libraries with index codes were generated using the TruSeq DNA PCR-Free Sample Prep Kit following manufacturer’s instructions, and assessed using the Qubit@ 2.0 Fluorometer and Agilent Bioanalyzer 2100 system for its quality. Then, the library was applied to paired-end sequencing (2 × 250 bp) using the Illumina Hiseq apparatus at Rhonin Biosciences Co., Ltd. (China). The sequences data were analyzed according to Usearch (http://drive5.com/uparse/) and QIIME pipeline [23]. Paired-end reads from the original DNA fragments were merged using FLASH [24], then, assigned to each sample according to the unique barcode. All reads with length less than 200 bp, average base quality scores less than 30, more than two ambiguous base ‘N’, and singletons were discarded [25]. Representative sequences were picked and potential chimera were removed using the Uchime algorithm [26]. Sequences were then clustered into operational taxonomic units (OTUs) at 97% identity threshold using UPARSE algorithms [27]. Taxonomy was named using the Silva database [28] and uclust classifier in QIIME. Representative sequences (OTUs) were aligned using PyNAST [29] embedded in QIIME. After quality checking, phylogenetic trees were reconstructed based on maximum likelihood–approximation method using the generalised time-reversible(GTR) model in FastTree [30].

### Community data analysis and statistics

Parameters in the demographics and clinical examination of all subjects were analyzed by t-test (age) or Chi-square test(gender). The OTUs in all samples were rarifies to give the same sequence number and analyzed by using R or Python (https://www.python.org/). Specifically, Simpson and Shannon index were used to investigate α-diversity among groups, and rarefaction curves were generated based on Mothur package to assess the current sequencing depth among all samples. The differences between OLPs and control was analyzed by t-test (pair-comparison). β-diversity analysis was estimated by Jaccard and PCoA based on unweighted_UniFrac distance using Permutational Multivariate Analysis of Variance (PERMANOVA) to identify whether there was significant difference among different groups. The statistics used for the comparison of micribiome at the compositional level including the analysis of relative abundance and core microbiome analysis between groups were Wilcoxon test and Duncan test. LDA effect size (LEFSE) [15] was assessed by using the Kruskal-Wallis rank sum test to detect bacterial component variation of different groups. Random forest analysis was applied to obtain the important indicator taxa using Random Forest package with 1000 trees and all default settings. Finally, the significant correlation of the difference on bacteria in all samples was analyzed by using Spearman’s rank correlation. Statistical comparisons of mean values for paired data and for data of more than two sets were performed with the Wilcoxon test and Kruskal test, respectively.

## Supplementary information

**Additional file 1: Figure S4.** The Box-plot of higher relative abundance in genus level. The ordinate represents the classification attribute, phylum, and genera level. The abscissa represents the reading abundance. The boxed line near the left of the vertical axis represents the lower quartile, which accounts for 25% of all values in the sample arranged in an ascending order. The vertical line of the box is the median, which accounts for 50% of all values in the sample arranged in an ascending order. The black square in picture shows the higher or lower relative abundance bacteria in E or NE OLP group.

**Additional file 2: Figure S7.** The Mean Decrease Gini Index in OLP and C groups. The abscissa on the left figure was the mean decrease Gini index, and the ordinate represented the bacteria classification. The right box plot was the relative abundance bacteria in three groups (***: *p* < 0.001; **: *p* < 0.01; *: *p* < 0.05).

## Data Availability

All Illumina squences data in our study were submitted to the NCBI Sequence Read Archive (SRA) under BioProject accession number PRJNA542018. All data generated or analyzed during this study are included in this published article.

## Abbreviations

OLP: Oral lichen planus
OUT: Operational taxonomic unit
PCoA: Principal Co-ordinates Analysis
PCR: Polymerase chain reaction
PerMANOVA: Permutational multivariate analysis of variance
RAU: Recurrent aphthous ulcerations
