# Supplementary figures and images for: Dysbiosis of saliva microbiome in patients with oral lichen planus
Source: BMC Microbiol. 2020 Apr 3;20:75. doi: 10.1186/s12866-020-01733-7 (PMC7118920; doi:10.1186/s12866-020-01733-7)

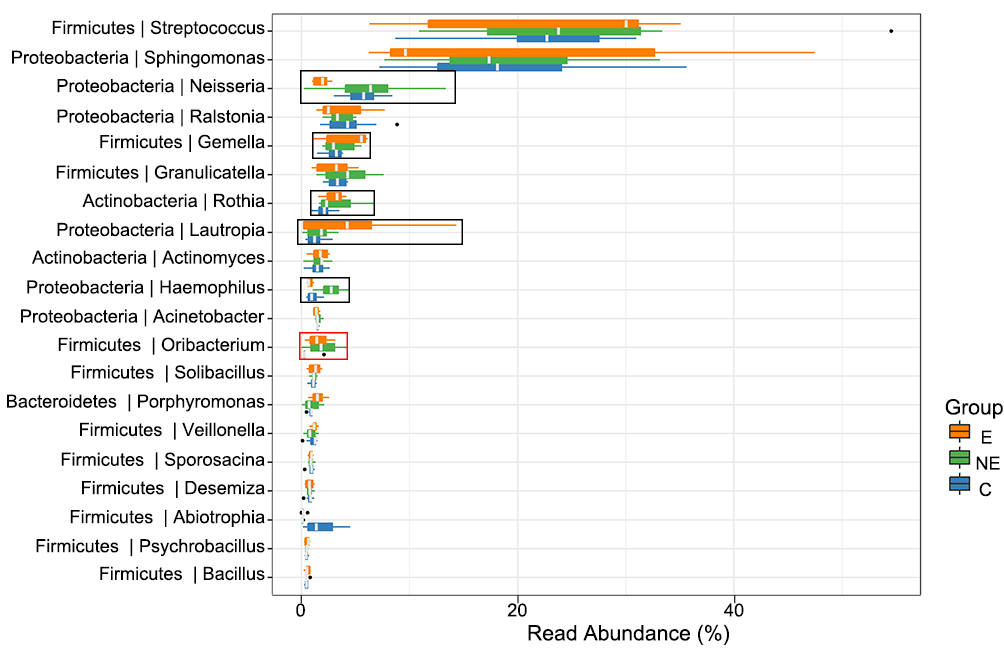

Supplement: Supplementary file 1 — Additional file 1: Figure S4. The Box-plot of higher relative abundance in genus level. The ordinate represents the classification attribute, phylum, and genera level. The abscissa represents the reading abundance. The boxed line near the left of the vertical axis represents the lower quartile, which accounts for 25% of all values in the sample arranged in an ascending order. The vertical line of the box is the median, which accounts for 50% of all values in the sample arranged in an ascending order. The black square in picture shows the higher or lower relative abundance bacteria in E or NE OLP group. [file 12866_2020_1733_MOESM1_ESM.zip › Fig 4S.tif]

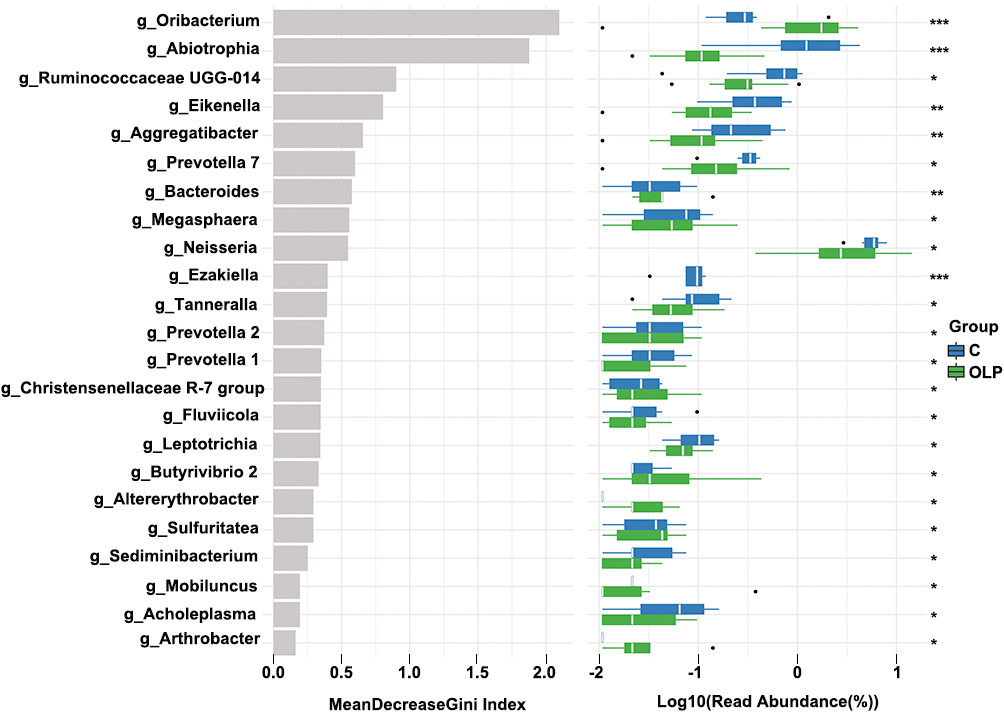

Supplement: Supplementary file 2 — Additional file 2: Figure S7. The Mean Decrease Gini Index in OLP and C groups. The abscissa on the left figure was the mean decrease Gini index, and the ordinate represented the bacteria classification. The right box plot was the relative abundance bacteria in three groups (***: p < 0.001; **: p < 0.01; *: p < 0.05). [file 12866_2020_1733_MOESM2_ESM.zip › Fig. 7S.tif]
